# Obligations to Advertisers

**Published:** 1916-05

**Authors:** 


### Obligations to Advertisers.

We wish to impress on our readers that this and practically every other periodical, medical or otherwise, monthly, weekly or daily, is largely supported by advertising. For instance, at present prices, the purchaser of a penny newspaper gets rather more than a cent's worth of paper at wholesale rates. It will thus be seen that, ultimately, current subscription rates for periodicals in general, can be maintained only if the reader responds to the advertisements. On the other hand, we wish it distinctly understood that in no case do we bring the pressure of the negative phase of this argument to bear upon prospective advertisers. So far as possible, we support our advertisers by asking a reasonable preference for them. In no case, do we use any methods that may work in any way to the detriment of non-advertisers. Under these circumstances, we feel that it is fair to ask that if, in any case, the reader prefers to deal with a non-advertiser, he should call the attention of the latter and of our advertising manager, to the benefits of medical journal publicity.

